# Changes to the identity of EndoC-βH1 beta cells may be mediated by stress-induced depletion of HNRNPD

**DOI:** 10.1186/s13578-021-00658-6

**Published:** 2021-07-23

**Authors:** Nicola Jeffery, David Chambers, Brandon M. Invergo, Ryan M. Ames, Lorna W. Harries

**Affiliations:** 1 Institute of Biomedical and Clinical Sciences, University of Exeter Medical School, Barrack Road, Exeter, EX2 5DW UK; 2 Kings College London, London, WC2R 2LS UK; 3 University of Exeter, Stocker Road, Exeter, EX4 4QJ UK; 4 University of Exeter, Stocker Road, Exeter, EX4 4QD UK

**Keywords:** Beta-cells, Cell differentiation, HNRNPD, RNA binding proteins

## Abstract

**Background:**

Beta cell identity changes occur in the islets of donors with diabetes, but the molecular basis of this remains unclear. Protecting residual functional beta cells from cell identity changes may be beneficial for patients with diabetes.

**Results:**

A somatostatin-positive cell population was induced in stressed clonal human EndoC-βH1 beta cells and was isolated using FACS. A transcriptomic characterisation of somatostatin-positive cells was then carried out. Gain of somatostatin-positivity was associated with marked dysregulation of the non-coding genome. Very few coding genes were differentially expressed. Potential candidate effector genes were assessed by targeted gene knockdown. Targeted knockdown of the *HNRNPD* gene induced the emergence of a somatostatin-positive cell population in clonal EndoC-βH1 beta cells comparable with that we have previously reported in stressed cells.

**Conclusions:**

We report here a role for the *HNRNPD* gene in determination of beta cell identity in response to cellular stress. These findings widen our understanding of the role of RNA binding proteins and RNA biology in determining cell identity and may be important for protecting remaining beta cell reserve in diabetes.

**Supplementary Information:**

The online version contains supplementary material available at 10.1186/s13578-021-00658-6.

## Background

The processes of life can produce a very stressful environment for cells. When the balance of homeostasis is disrupted, cellular stressors such as fluctuating glycaemia, dyslipidaemia, hypoxia or increased levels of inflammatory factors may become so prevalent that the viability or identity of cell populations may become compromised. This phenomenon occurs in multiple tissues, but is particularly prevalent in tissues such as pancreatic islets that are directly involved in metabolic homeostasis [1]. Loss of beta cell mass is a characteristic of both type 1 and type 2 diabetes (T1D and T2D) [2], arising from apoptosis, but also from changes in endocrine cell identity [3]. The consequences of these changes are a progressive deterioration in the ability of the pancreas to produce enough insulin to regulate the blood sugar. However, even in long duration diabetes, a small reservoir of active and responsive beta cells remains. Even many years after diagnosis, the majority of patients with T1D maintain glucose responsive post-prandial insulin secretion [4]. The protection of these ‘stress resistant’ beta cells is a key clinical priority, because in the future, strategies for their regeneration may emerge.

Beta cell identity is maintained by several mechanisms. Firstly, cells are programmed to remain as beta cells, even in the face of cellular stress, by the expression of a portfolio of beta cell transcription factors, which include *PDX1, NKX6-1, PAX6, NKX2-2, MAFA* and FOXO1 [5, 6]. Beta cell identity is also maintained by the exclusion of expression of genes associated with other endocrine cell types with non-oxidative metabolism (‘disallowed’ genes). These include genes encoding lactate dehydrogenase (*LDHA*) and the monocarboxylate transporter 1 (*SLC16A1*) [7]. Under conditions of cell stress, beta cells may undergo transdifferentiation, de-differentiation and/or re-differentiation into other pancreatic endocrine cell types [8–11], although the relevance of this to human disease remains to be established. Data in humans are more scarce, but dedifferentiation changes have been documented [12] as have cell identity changes from beta cells to alpha cells [13] or to delta cells [14].

We have previously determined that exposure to diabetomimetic cellular stressors leads to the emergence of a small somatostatin-positive population in the clonal human beta cell line EndoC-βH1 [14]. This is echoed in our observation of elevated numbers of somatostatin-positive cells in the pancreatic islets of patients with either T1D or T2D [14]. Transcriptomic analysis of mixed cultures revealed that transcripts encoding proteins involved in maintenance of beta cell fate or function demonstrated disruption, as did transcripts involved in the regulation of mRNA splicing, and splicing patterns themselves. These changes were ablated by the removal of the cellular stressor, or by treatment with the AKT inhibitor SH-6 [14]; AKT is known to be a negative regulator of splicing factor expression [15]. Whilst these findings represented a useful step forward in our understanding of how diabetes-related cellular stressors may mediate beta cell identity changes, our understanding of this process is still incomplete. The gene expression changes occurring specifically in the somatostatin-positive cells are difficult to deduce from mixed cultures, since some changes may be induced by cell stress, but are unrelated to changes in hormone expression.

Here, we aimed to identify the gene expression changes, and the specific effector genes, that differentiate the somatostatin-positive population from the somatostatin-negative cell population under conditions of cellular stress. We cultured human clonal beta cell line EndoC-βH1 in the presence of a diabetomimetic environment (25 mM glucose, 50 μM palmitic acid), and isolated the emergent somatostatin-positive cell population by fluorescence assisted cell sorting (FACS). Differential gene expression in somatostatin-positive cells and somatostatin-negative counterparts was assessed in the same culture using differential gene expression analysis, weighted gene network correlation analysis (WGCNA) and GO terms enrichment analysis. We determined that the major feature of the somatostatin-positive cell population was a dramatic dysregulation of the non-coding genome. Of the 100 most dysregulated transcripts, only nine originated from coding genes. Knockdown of key coding and non-coding transcripts disrupted in somatostatin-positive cells revealed that ablation of the expression of the heterogeneous nuclear ribonucleoprotein particle D (*HNRNPD*) gene alone resulted in the emergence of a similar somatostatin-positive population of cells to that seen in our original work. The *HNRNPD* gene encodes a multifunctional RNA binding protein with known roles in the regulation of the non-coding genome, regulation of mRNA splicing, stability and translation efficiency [16–18]. HNRNPD is one of a portfolio of RNA binding proteins that are associated with cellular plasticity and response to cellular stress [19, 20]. Our data are consistent with a model by which stress-related changes in *HNRNPD* gene expression levels lead to transcriptome-wide changes to the dynamics of RNA regulation, dysregulation of the non-coding genome and the emergence of a somatostatin-positive cell population.

## Results

### Isolation of a somatostatin-positive subpopulation of EndoC-βH1 cells

**Fig. 1:**
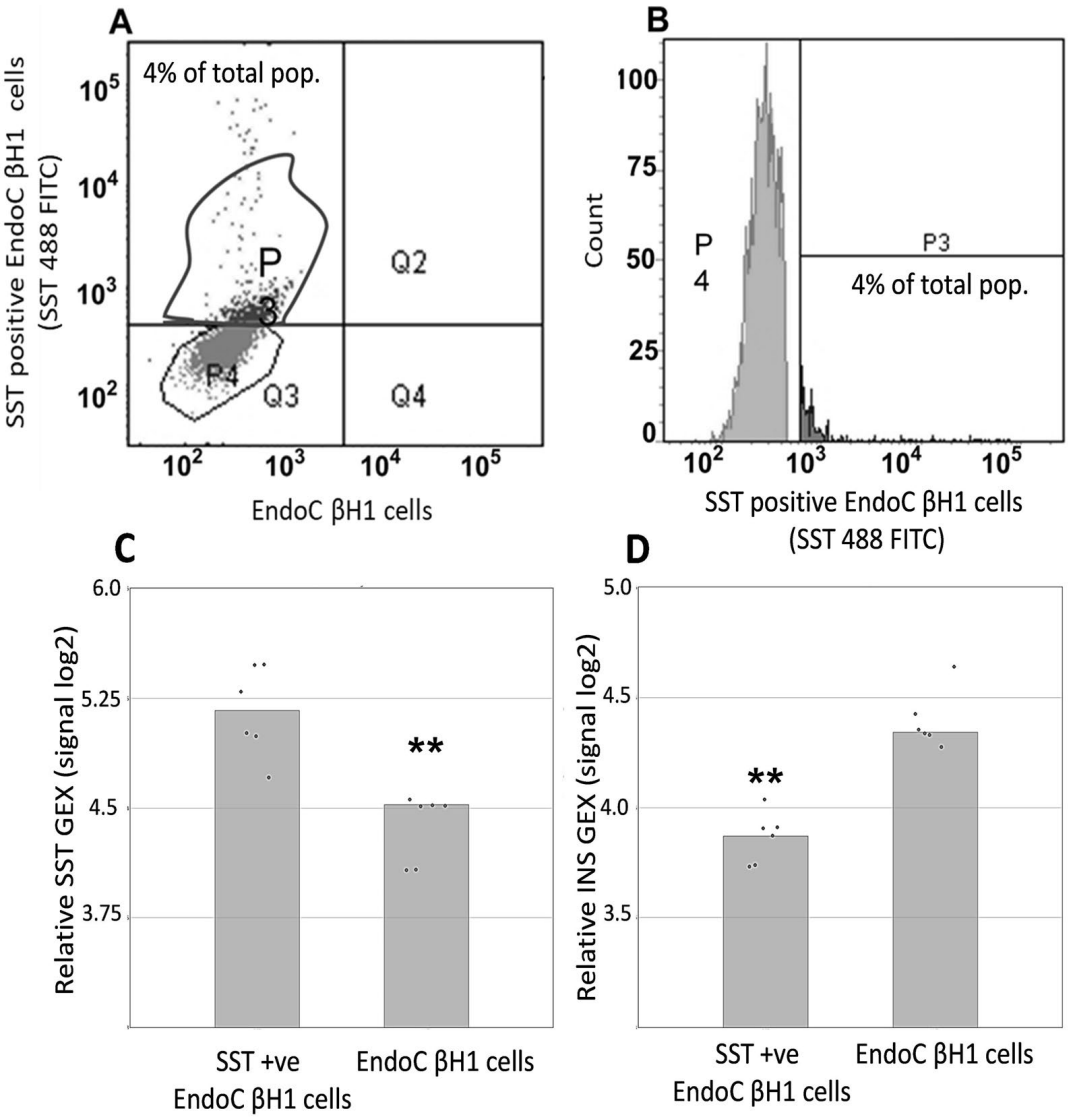
Isolation of an enriched population of somatostatin-positive cells. We isolated an enriched population of somatostatin-positive cells by FACS (n = 6 biological replicates; 20,000 cells per replicate). Figure shows ungated EndoC βH1 cells that were stained and sorted by SST expression. Gating thresholds were set using unstained cells as a control and an isotype control. **A** Graph shows gating around the P3 FITC somatostatin-positive subpopulation. The P4 gate is surrounding those cells with no somatostatin staining. **B** Graph shows gating around the P3 FITC somatostatin-positive sub population. The gate was set conservatively to ensure a more pure enrichment of somatostatin-positive cells. **C** Graph shows log2 increase in the expression of somatostatin (*SST*) in the enriched population of somatostatin-positive beta cells. **D** Graph shows log2 decrease in the expression of insulin (*INS*) in the enriched population of somatostatin-positive beta cells. **p =  < 0.01

Treatment with 25 mM glucose and 50 μM palmitic acid induced the emergence of a somatostatin-positive sub-population of EndoC-βH1 cells comprising approximately 4% of the population, concordant with our previous observations [16]. An additional 1–2% of cells demonstrated evidence of dual hormone positivity for somatostatin and insulin, but it is currently unclear whether these cells are genuinely dual hormone positive, or instead represent ‘bleed through’ of fluorophores or overlap of cells in different planes. We were able to isolate an enriched population of approximately 20,000 cells per replicate for both somatostatin-enriched and somatostatin-depleted cell populations by FACS (Fig. 1a, b). Quantitative real-time PCR analysis revealed that the somatostatin-positive cells demonstrated a 2.5 fold increase in somatostatin gene expression (*p* = 0.003; Fig. 1c) and a 2.7 fold decrease in insulin expression (*p* = 0.007; Fig. 1d). There was no change in the expression of delta cell specific genes *HHEX* and *GHSR* (*p* = 0.71 and 0.75 respectively)*.* Although not a pure population consisting exclusively of somatostatin-positive cells, we achieved a suitable degree of enrichment to be able to assess any transcriptomic changes.

### Few coding genes demonstrate differential expression in somatostatin-positive cells

Our initial analysis identified 837 differentially-expressed genes, out of 38,623 genes expressed in either insulin or somatostatin-positive cells. 33 of these demonstrated > twofold differences in expression, although none reached statistical significance after adjustment for multiple testing (Table 1). It is particularly noteworthy that only 21 of these transcripts in the top 100 represent coding genes. These were *HNRNPD* (fold change − 2.7, unadjusted p value = 0.0005), *GPN1* (fold change 2.35; p = 0.002), *PHF12* (fold change − 2.29; p value = 0.0002), *C1Orf123* (fold change − 2.18; p = 0.002), *ZNF248* (fold change = − 2.08; p = 0.0004), *SENP7* (fold change − 2.05; p = 0.0004), *DNAJC11* (fold change − 2.00; p = 0.001) and *MALT1* (fold change 2.00; p = 0.001). Another prominent feature in this dataset is the prevalence of differentially-expressed small RNAs, processed pseudogene derived transcripts, variant U6 and U7 small nucleolar ribonucleoparticles (snRNPs), snoRNAs and other non-coding transcripts in somatostatin-positive cells.

**Table 1:** This table shows the 100 most dysregulated genes with a p value of < *p* = 0.005

Gene symbol	INS Avg (log2)	SST Avg (log2)	INS Standard deviation	SST Standard deviation	Fold change	p-val
RNU7-26P	6.85	4.43	1.83	0.21	− 5.33	0.00110
RNU7-53P	8.17	6.02	1.98	0.75	− 4.45	0.00270
MIR3198-1	8.15	6.1	1.34	0.67	− 4.15	0.00400
MTND4LP1	8.84	7.01	0.93	0.4	− 3.56	0.00010
MIR548T	5.87	4.04	1.22	0.21	− 3.55	0.00040
***MIR181A1***	***3.75***	***5.4***	***0.43***	***0.83***	***3.16***	***0.00100***
SNORA41	9.39	7.76	0.94	0.26	− 3.1	0.00040
LINC01628	4.49	6.08	0.45	0.73	3.02	0.00040
SNRPGP11	7.47	5.92	0.76	0.58	− 2.93	0.00070
RNU6-866P	8.7	7.17	0.45	0.58	− 2.9	0.00010
MED6P1	5.76	4.26	0.8	0.34	− 2.83	0.00470
**HNRNPD**	**5.45**	**4.01**	**0.46**	**0.46**	− **2.7**	**0.00050**
RNU2-36P	5.93	4.5	0.48	0.47	− 2.69	0.00007
SNORA46	6.99	5.58	1.14	0.22	− 2.65	0.00200
MIR373	7.24	5.88	0.33	0.23	− 2.57	0.00002
RNA5-8SP3	5.41	4.09	0.57	0.43	− 2.51	0.00170
RNU7-24P	6.27	4.94	0.85	0.36	− 2.51	0.00180
MIR1277	6.21	4.88	0.54	0.83	− 2.5	0.00100
YWHAZP5	6.06	4.75	0.63	0.42	− 2.49	0.00040
MIR548AC	12.26	10.97	1.3	0.52	− 2.45	0.00270
**GPN1**	**3.77**	**5**	**0.32**	**0.61**	**2.35**	**0.00200**
MIR548F5	7.01	5.81	0.71	0.6	− 2.31	0.00280
**PHF12**	**8.04**	**6.84**	**0.62**	**0.58**	− **2.29**	**0.00180**
RNY4P37	5.13	3.94	0.62	0.2	− 2.28	0.00007
RAB11AP1	4.04	5.18	0.46	0.79	2.2	0.00270
**C1orf123**	**6.23**	**5.1**	**0.74**	**0.68**	− **2.18**	**0.00220**
RN7SL748P	5.53	4.42	0.71	0.57	− 2.15	0.00360
ZNF965P	5.81	4.71	0.38	0.44	− 2.13	0.00470
MTND1P23	18.75	17.67	0.85	0.81	− 2.12	0.00230
RN7SKP146	6.26	5.18	0.63	0.4	− 2.12	0.00400
RNU7-188P	5.43	4.35	0.76	0.32	− 2.11	0.00250
MTND3P3	5.9	4.82	0.45	0.42	− 2.11	0.00260
POLR3KP1	4.08	5.15	0.24	0.31	2.09	0.00007
RNU6-250P	3.82	4.89	0.24	0.51	2.09	0.00110
**ZNF248**	**5.68**	**4.63**	**0.23**	**0.43**	− **2.08**	**0.00040**
**SENP7**	**4.92**	**3.89**	**0.58**	**0.17**	− **2.05**	**0.00040**
MIR5684	8.43	7.4	0.65	0.67	− 2.04	0.00270
RNU1-133P	5.7	4.68	0.7	0.46	2.04	0.00360
MIR5003	6.02	4.99	1.15	0.44	2.04	0.00430
GXYLT1P5	4.3	5.32	0.43	0.62	2.03	0.00230
RNU6-882P	3.95	4.95	0.28	0.36	2.01	0.00180
**DNAJC11**	**4.75**	**3.76**	**0.68**	**0.25**	− **2**	**0.00100**
RNU6-571P	5.97	4.96	0.45	0.3	− 2	0.00120
RPS12P2	6.08	5.08	0.45	0.59	2	0.00290
**MALT1**	**3.79**	**4.78**	**0.21**	**0.76**	**2**	**0.00330**
RNY4P34	7.19	6.2	0.43	0.46	− 1.99	0.00070
RNU7-41P	4.97	3.99	0.45	0.19	− 1.97	0.00050
SCARNA11	6.47	5.5	0.5	0.38	1.97	0.00160
GAPDHP28	4.7	3.72	0.68	0.11	1.97	0.00190
FTH1P7	7.46	6.48	0.3	0.53	1.97	0.00210
MEOX2-AS1	4.41	5.38	0.27	0.77	1.96	0.00250
U7	4.21	5.17	0.41	0.41	1.95	0.00270
RNU1-32P	6.47	5.52	0.78	0.25	− 1.94	0.00320
RNU6-966P	5.49	4.54	0.6	0.26	− 1.94	0.00400
LAPTM4BP2	4.02	4.97	0.29	0.56	1.94	0.00130
CSHL1	4.01	4.96	0.19	0.65	1.94	0.00470
ZBTB20-AS4	4.1	5.05	0.3	0.47	1.93	0.00180
OMG	4.25	5.19	0.42	0.4	1.92	0.00140
IGLV3-15	6.04	5.1	0.46	0.16	− 1.91	0.00360
MTCO1P3	5.71	4.77	0.38	0.42	− 1.91	0.00410
CA5BP1	6.98	6.06	0.48	0.28	− 1.9	0.00100
UQCRHP4	4.75	3.83	0.34	0.22	− 1.89	0.00040
MTCO3P12	15.24	14.32	0.76	1.12	− 1.89	0.00170
MTND6P11	8.08	7.16	0.55	0.85	− 1.89	0.00170
FAR1P1	5.21	4.29	0.42	0.35	− 1.89	0.00230
RPL30P15	3.93	4.85	0.36	0.46	1.89	0.00430
ATXN1	7.17	6.26	0.23	0.45	− 1.88	0.00060
MIR450A1	4.47	5.38	0.43	0.28	1.88	0.00100
HMGN2P31	6.09	5.18	0.23	0.26	− 1.87	0.00010
HMGN2P31	6.09	5.18	0.23	0.26	− 1.87	0.00010
RPL21P32	4.99	4.09	0.37	0.31	− 1.87	0.00270
RN7SKP94	7.73	6.84	0.41	0.37	− 1.86	0.00100
***MIR543***	***4.89***	***4.07***	***0.5***	***0.38***	− ***1.76***	***0.00140***
HMGN2P15	6.99	6.1	0.61	0.18	− 1.85	0.00140
HNRNPH3	5.73	4.84	0.38	0.34	− 1.85	0.00180
TCTA	7.11	6.23	0.59	0.44	− 1.85	0.00440
IKZF3	4.87	3.99	0.47	0.13	− 1.84	0.00080
SUMO2P15	5.17	4.29	0.41	0.46	− 1.84	0.00300
UBDP1	5.17	4.3	0.45	0.39	− 1.83	0.00360
FAM81B	3.89	4.76	0.33	0.27	1.83	0.00050
DHX9	4.09	4.97	0.34	0.55	1.83	0.00350
HERC2P8	5.21	4.35	0.42	0.15	− 1.82	0.00020
RNU6-139P	5.24	4.38	0.49	0.37	− 1.82	0.00070
ULK4P3	6.46	5.6	0.34	0.38	− 1.82	0.00150
MIR450B	4.95	4.08	0.42	0.27	− 1.82	0.00220
C3orf35	4.92	4.06	0.5	0.21	− 1.82	0.00480
OTUD3	5.15	4.3	0.48	0.25	− 1.81	0.00200
RPL36AP48	3.94	4.8	0.16	0.41	1.81	0.00190
FAM157C	4.96	4.12	0.42	0.25	− 1.8	0.00120
MTCO1P5	5.1	4.26	0.3	0.2	− 1.79	0.00220
FAM177B	4.69	3.85	0.59	0.18	− 1.79	0.00250
RNU6-903P	4.04	4.88	0.4	0.35	1.79	0.00190
HMGN1P24	5.06	4.23	0.36	0.15	− 1.78	0.00060
TARDBPP1	5.55	4.72	0.68	0.15	− 1.78	0.00200
DCDC5	4.42	3.59	0.51	0.28	− 1.78	0.00490
MTCO3P22	5.56	4.73	0.47	0.27	− 1.77	0.00340
LINC00243	4.51	3.69	0.32	0.34	− 1.76	0.00330
PRKG2	5.17	4.36	0.56	0.29	− 1.76	0.00460
COX6CP17	6.36	5.56	0.4	0.24	− 1.75	0.00300
RN7SKP177	6.8	6	0.35	0.22	− 1.74	0.00030

Pre-microRNAs demonstrating statistical significant changes in somatostatin-positive cells at the level of *p* =  < 0.001 are highlighted in bold and italics. Coding genes (canonical transcripts not pseudogene variants) with fold changes > 2.0 or < − 2.0 are highlighted in bold

### The differentially-expressed genome in somatostatin-positive cells is enriched in non-coding transcripts

Having observed that many of the most dysregulated transcripts in our dataset were non-coding transcripts, we carried out an enrichment analysis to identify whether the apparent over-representation of this class of genes was greater than would be expected by chance. This analysis confirmed that non-coding multiple complex loci, small RNAs, single gene non-coding loci and precursor miRNAs all demonstrated significant over-representation in our data (p = 4.58 × 10^–7^, p = 1.08 × 10^–12^, p = 6.41 × 10^–9^ and p = 3.16 × 10^–8^ respectively, whilst coding genes demonstrated an under-representation (p = 1.40 × 10^–9^; Table 2). The ribosomal class showed no significant change in the number of expected dysregulated genes (*p* = 0.07).

**Table 2:** Non-coding transcripts are over-represented in somatostatin-positive cells

	Non-coding genes observed in SST positive cells	Non-coding genes expected in SST positive cells	p value
Coding genes	134	216	2.05 × 10^–9^
Non-coding genes	636	519	4.33 × 10^–9^
Precursor miRNAs	118	71	5.76 × 10^–9^
Small RNAs	220	137	8.09 × 10^–14^

We carried out a χ^2^ analysis to determine whether there were more non-coding transcripts in somatostatin-positive cells than one would expect by chance. This table shows the results of this analysis for coding transcripts, non-coding transcripts, precursor miRNAs and small RNAs

### WGCNA suggests that transcripts enriched in somatostatin-positive cells cluster into modules associated with dysregulated ubiquitination, RNA and microRNA processing

We then looked for modules of co-ordinated differential expression in somatostatin-positive cells by the use of a weighted gene correlation network analysis (WGCNA). After initial filtering of the somatostatin-positive cell dataset to remove AceView genes and unannotated transcripts, 52,986 gene expression features remained in our dataset which were organised into 227 modules. WGCNA identified 11 nominally-significant gene clusters, although again, these did not meet the threshold for multiple testing (Table 3a). The top three eigengene modules (light sky blue 2, orange red 4 and salmon 1) were taken forward into Panther GO biological processes analyses to identify biochemical or cellular function pathways which were enriched in modules of genes demonstrating differential expression in somatostatin-positive cells. From the light sky blue 2 module 11 biological process pathways passed a correction for false discovery rate and identified enrichment for processes relating to dysregulated proteostasis (Table 3b). The top three processes included protein modification by small protein removal (p = 3.51 × 10^–6^), protein deubiquitination (*p* = 4.22 × 10^–6^) and modification-dependent macromolecule catabolic processes (2.86 × 10^–4^; Table 3b). GO biological processes for the orange red 4 and salmon 1 gene modules showed nominal significance for nine biological processes for which the top three were miRNA 2ʹ-*O*-methylation (*p* = 0.003), immune system process (*p* = 0.004) and negative regulation of pre-miRNA processing (*p* = 0.005; Table 3b).

**Table 3:** Gene clusters from WGCNA analysis and GO terms analysis of genes within the top three clusters

(A) WGCNA				
Eigengene module	LogFC	p	Adj p	B
ME light sky blue2	− 0.46265489	0.012	0.436	− 4.00
ME orange red 4	− 0.461540016	0.013	0.436	− 4.010
ME salmon 1	− 0.428245371	0.020	0.436	− 4.11
ME indian red 1	0.418718931	0.023	0.436	− 4.14
ME alice blue	0.414679702	0.025	0.436	− 4.5
ME pink 3	0.408739235	0.027	0.436	− 4.16
ME wheat 3	− 0.395082849	0.032	0.436	− 4.20
ME sky blue 2	0.384220441	0.037	0.436	− 4.23
ME indian red 2	− 0.380769172	0.039	0.436	− 4.24
ME medium purple	− 0.380324742	0.039	0.436	-4.24
ME blue 2	− 0.377223329	0.041	0.436	-4.25

B			
GO biological process complete	Fold enrichment	Raw *p value*	FDR
Protein modification by small protein removal (GO:0070646)	20.27	4.42 × 10^–10^	3.51 × 10^–6^
Protein deubiquitination (GO:0016579)	21.51	2.66 × 10^–10^	4.22 × 10^–6^
Modification-dependent macromolecule catabolic process (GO:0043632)	10.83	9.03 × 10^–8^	2.86 × 10^–4^
Modification-dependent protein catabolic process (GO:0019941)	11.05	7.63 × 10^–8^	3.03 × 10^–4^
Ubiquitin-dependent protein catabolic process (GO:0006511)	11.2	6.84 × 10^–8^	3.62 × 10^–4^
Proteolysis involved in cellular protein catabolic process (GO:0051603)	9.98	1.79 × 10^–7^	4.73 × 10^–4^
Cellular protein catabolic process (GO:0044257)	9.46	2.79 × 10^–7^	6.33 × 10^–4^
Protein catabolic process (GO:0030163)	8.72	5.51 × 10^–7^	0.001
Macromolecule catabolic process (GO:0009057)	6.32	2.05 × 10^–6^	0.004
Cellular macromolecule catabolic process (GO:0044265)	6.52	5.88 × 10^–6^	0.009
Protein modification by small protein conjugation or removal (GO:0070647)	5.83	1.44 × 10^–5^	0.021
MiRNA 2ʹ-*O*-methylation (GO:0061715)	> 100	0.003	
Immune system process (GO:000276)	3.15	0.004	
Negative regulation of pre-miRNA processing (GO:2000632)	> 100	0.005	
Regulation of pre-miRNA processing (GO:2000631)	> 100	0.005	
Entry of viral genome into host nucleus through nuclear pore complex via importin (GO:0075506)	> 100	0.005	
‘De novo’ co-translational protein folding (GO:0051083)	> 100	0.005	
Intramembranous ossification (GO:0001957)	> 100	0.005	
Direct ossification (GO:0036072)	> 100	0.005	
Osteoclast fusion (GO:0072675)	> 100	0.005	

(A) WGCNA: The table illustrates the 11 gene modules identified by WGCNA and reaching statistical significance after multiple testing that demonstrate correlated differential expression in somatostatin-positive cells. The table gives the average module logged fold change (logFC), the logged odds ratio that the module is differentially expressed (B) and the nominal p value (p) and the FDR-adjusted p value (Adj p). (B) GO analysis: The table gives the Gene Ontology (GO) biological processes pathway, the fold enrichment of differentially-regulated genes within that pathway and the p value for significance for biological processes pathways that contain significantly more differentially regulated gene modules in somatostatin-positive cells than would be expected by chance. Only GO processes from the ‘light sky blue 2’ gene module made FDR corrected *p* values, genes from modules ‘Orange Red 4’ and ‘Salmon 1’ were nominally significant

### Patterns of differential splicing in somatostain positive cells

We assessed differential expression at the level of exon usage in somatostatin-positive cells compared with somatostatin-negative cells. In the dataset overall, we have identified that approximately 26% of all splice events in somatostatin-positive cells demonstrate disrupted patterns of mRNA processing. These changes demonstrated dysregulation of constitutive splicing in the form of intron retentions (55.74%), alternative 3ʹ acceptor site usage (25.59%) and alternative 5ʹ donor site usage (19.67%). Genes with differential splicing patterns are given in Table 4a. Genes were screened for passing nominal significance of *p* < 0.01, a splicing index score of > 2 or < − 2 and an exon event score of > 0.1. Both splicing index and exon event scores are algorithms within the TAC analysis software that provides a measure for likelihood of a splicing event. Splicing index scores of > 2 or < − 2 indicate a possible alternative splicing event [21]. Exon events are scored between 0 and 1 with events > 0.1 an indicator for an aberrant splicing event [21]. The screen identified 29 genes (Table 4a) which were taken forward to Panther for GO Biological Processes analysis and identified nominal significance (p < 0.01) for 21 different biological process pathways, of which the top was positive regulation of transcription from RNA polymerase II promoter in response to calcium ion (Table 4b).

**Table 4:** Differentially spliced genes in somatostatin-positive cells

A					
Location of splice event	Gene symbol	Exon Splicing Index	Exon p-val	Exon event name	Exon event score
Exon 7/8	DUS4L	− 4.02	0.0082	Alternative 3ʹ acceptor site	0.2
Exon 6/6	CLIC4	5.34	0.0015	Alternative 5ʹ donor site	0.2
3ʹ end of exon 1/1	RNU5B-6P	− 3.93	0.0087	Alternative 5ʹ donor site	0.2
Exon 11/11	DBNL; MIR6837	− 4	0.0049	Alternative 5ʹ donor site	0.21
Exon 7/7	NDUFS3	− 4.38	5.35E−05	Alternative 5ʹ donor site	0.21
Exon 6/6	ARHGDIB	4.46	0.0077	Alternative 5ʹ donor site	0.21
Exon 20/24	GTF2IP1	4.64	9.32E−05	Alternative 3ʹ acceptor site	0.21
Exon 3/6	TRIM64C	5.18	0.0089	Alternative 3ʹ acceptor site	0.21
Intron 4/12	NFKBIZ	5.34	0.0028	Alternative 5ʹ donor site	0.21
Exon 1/3	VAX1	9.05	0.0038	Alternative 3ʹ acceptor site	0.21
Intron 8/15	CARF	− 2.71	0.0014	Intron retention	0.21
Exon 6/6	HMGN1	7.78	0.0017	Alternative 3ʹ acceptor site	0.21
Intron 11/36	NBPF12	2.68	0.0023	Intron retention	0.21
Intron 6/35	NUP160	3.74	0.0023	Intron retention	0.22
Intron 2/9 or Intron 1/8	AP3M2	4.34	0.0051	Intron retention	0.22
Alternative 3ʹ acceptor site	COL28A1	4.29	0.0068	Alternative 3ʹ acceptor site	0.23
Intron 12/21	ACAP1	3.01	0.0042	Intron retention	0.23
Exon 3/5	HINT2	2.95	0.0004	Intron retention	0.24
Intron 11/12	SEPT7P2	− 3.01	0.0089	Intron retention	0.24
Intron 3/8 or Intron 2/7	ACTG2	− 3.09	0.005	Intron retention	0.24
Intron 6 in exon 7/16	SLC9A8	14.06	0.0043	Intron retention	0.25
Intron1 in exon 1/5	CUTA	3.34	0.0059	Intron retention	0.27
Intron 14/29 or 30	TRAPPC11	− 10.49	0.0014	Intron retention	0.32
Intron 6/8	STAG3L3; STAG3L2	3.78	0.0002	Intron retention	0.32
Intron 3/11 or Intron 2/10	ACVR2A	5.65	0.0077	Intron retention	0.33
Intron 5/8	PRKRIP1	4.3	0.0019	Intron retention	0.36
Intron 5/12 or Intron 7/13 or Intron 4/9	PHYKPL	20.83	0.0014	Intron retention	0.36
Intron 12/18 or Intron 13/19	MPP3	− 4.93	0.0013	Intron retention	0.37
Intron 4/7	GLT1D1	− 4.16	0.0004	Intron retention	0.37

B		
GO biological process complete	Fold enrichment	Raw *p* value
Positive regulation of transcription from RNA polymerase II promoter in response to calcium ion (GO:0061400)	> 100	0.00
Positive regulation of NAD + ADP-ribosyltransferase activity (GO:1901666)	> 100	0.01
Regulation of NAD + ADP-ribosyltransferase activity (GO:1901664)	> 100	0.01
Post-embryonic camera-type eye morphogenesis (GO:0048597)	> 100	0.01
Penile erection (GO:0043084)	> 100	0.01
Cellular response to redox state (GO:0071461)	> 100	0.01
Pyrimidine dimer repair by nucleotide-excision repair (GO:0000720)	> 100	0.01
Sertoli cell proliferation (GO:0060011)	> 100	0.01
Constitutive secretory pathway (GO:0045054)	> 100	0.01
Mesenchyme migration (GO:0090131)	> 100	0.01
tRNA dihydrouridine synthesis (GO:0002943)	> 100	0.01
Negative regulation of trophoblast cell migration (GO:1901164)	> 100	0.01
Sperm ejaculation (GO:0042713)	> 100	0.01
Post-embryonic eye morphogenesis (GO:0048050)	> 100	0.01
Post-embryonic camera-type eye development (GO:0031077)	> 100	0.01
Negative regulation of neuroblast proliferation (GO:0007406)	99.01	0.01
Positive regulation of activin receptor signaling pathway (GO:0032927)	99.01	0.01
Positive regulation of T-helper 17 cell differentiation (GO:2000321)	99.01	0.01
Pyrimidine dimer repair (GO:0006290)	88.01	0.01
Post-embryonic animal organ morphogenesis (GO:0048563)	88.01	0.01
Positive regulation of T-helper 17 type immune response (GO:2000318)	79.21	0.01

(A) Differentially spliced genes: This table shows the 29 most differentially spliced genes with exon *p* value of <*p* = 0.01, splicing index of > 2 or < −2 and exon event score of > 02. Splicing index is used as a measure to detect alternative splicing events where a value larger or smaller than one indicates the presence of alternative splicing. Exon event score is a further measure of an aberrant or alternative splicing events. (B) GO terms analysis: The table gives the Gene Ontology (GO) biological processes pathway, the fold enrichment of differentially-regulated genes within that pathway and the *p* value for significance for biological processes pathways that contain significantly more differentially spliced genes in somatostatin-positive cells than would be expected by chance

### Validation of differentially expressed miRNA targets

The associations we have identified are nominal, and do not survive adjustment for multiple testing, but do highlight some interesting candidates for follow up. The quantity and quality of RNA that can be obtained from cells following cell sorting with an internal marker precludes subsequent validation of many differentially-regulated transcripts; RNA produced from these experiments is likely to be fragmented and unlikely to contain fragments of > 50 bp. We were able to assess levels of mature miRNAs in the sample set, which were suitable for analysis on the basis of their small size of ~ 25 bp. The other classes of small RNA (variant snRNP components, snoRNAs) are of the order of 150 bp, and as such not likely to be accessible in the RNA samples by qRTPCR. We identified nine pre-microRNAs with a *p* value of < 0.001 in our initial list of potentially dysregulated transcripts (miR-373, miR-543, miR-548t, miR-181a-3p, miR-181a-5p, miR450a-1-3p, miR-450a-5p, miR-6874-3p and miR-6874-5p). The mature forms of these pre-microRNAs were assayed by qRT-PCR using RNA from sorted somatostatin-positive and somatostatin-negative EndoC-βH1 cell populations. Of these, only the mature forms of miR-543 and miR-181a-5p demonstrated dysregulated expression in somatostatin-positive cells (miR-543 downregulation *p* = 0.001 and miR-181a-5p upregulation *p* = 0.001; Additional file 1: Table S1).

### miRNAs miR-181a-5p and miR-543 do not influence changes in EndoC-βH1 cell identity

**Fig. 2:**
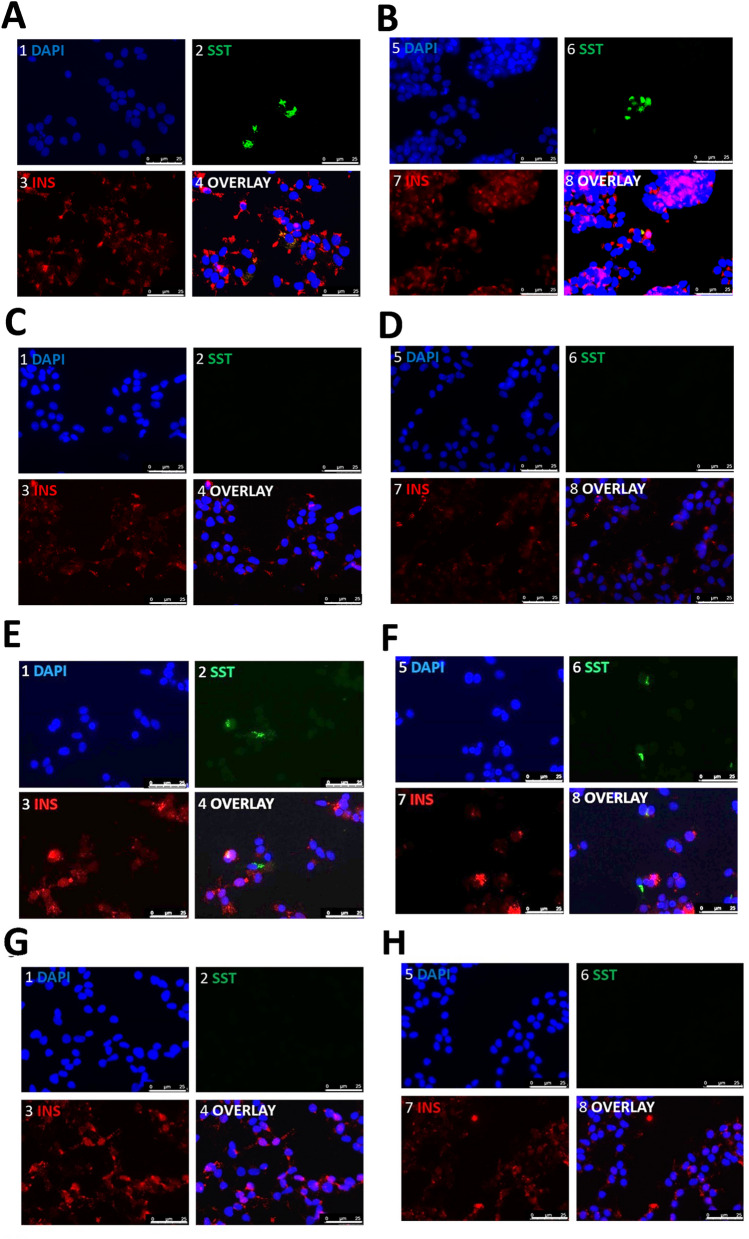
miR-543 and miR-181a-5p mimics and inhibitors do not induce/rescue somatostatin positivity in EndoC-βH1 cells. This figure illustrates the hormone profiles of EndoC-βH1 cells following manipulation of miR-543 or miR-181a levels and analysed using ANOVA. All data are derived from three biological replicates. **A** Stressed (25 mM glucose) EndoC-βH1 cells treated with miR-543 mimic. **B** EndoC-βH1 cells treated with 25 mM glucose only. **C** Non-stressed cells (in standard culture conditions) treated with an antagomiR to miR-543. **D** Non-stressed control (standard culture conditions) EndoC-βH1 cells. **E** Stressed (25 mM glucose) EndoC-βH1 cells treated with miR-181a-5p antagomiR. **F** EndoC-βH1 cells treated with 25 mM glucose only. **G** Non-stressed cells (standard culture conditions) treated with a miR-181a-5p mimic. **H** Non-stressed (standard culture conditions) control EndoC-βH1 cells. Cell nuclei are labelled with DAPI (blue). Somatostatin (SST) expression is given in green. Insulin (INS) expression is given in magenta. Scale bars represent 0–25 μM

To assess the role of the two mature miRNAs demonstrating evidence of altered expression in somatostatin-positive enriched cell populations (miR-181a-5p and miR-543), we altered their expression using miRNA mimics and antagomiRs in the presence and absence of 25 mM glucose and 50 μM Palmitic acid. We then assessed whether our intervention was able to induce the emergence of a somatostatin-positive population of cells in unstressed cells, or to protect against the emergence of a somatostatin-positive cell population in stressed cells when miR levels were adjusted to those seen in stressed and non-stressed cells respectively. Changes in miRNA levels after mimic or antagomiR treatment are provided in Additional file 1: Table S2. Treatment with 25 mM glucose and 50 µM palmitic acid induced the emergence of a similar somatostatin-positive cell population to that we have previously observed (~ 5% of the population), but co-treatment with the miR-543 mimic and miR-181a-5p inhibitor did not abrogate the expression of somatostatin following immunofluorescence characterisation of hormone profiles. Cells similarly transfected with the miR-543 inhibitor and miR-181a-5p mimic but not exposed to additional cell stresses also showed no changes to their hormone profiles upon immunofluorescent characterisation (see Fig. 2 and Additional file 1: Figure S1).

### HNRNPD gene knockdown causes the emergence of a somatostatin-positive population of EndoC-βH1 ‘delta-cell like’ cells

**Fig. 3:**
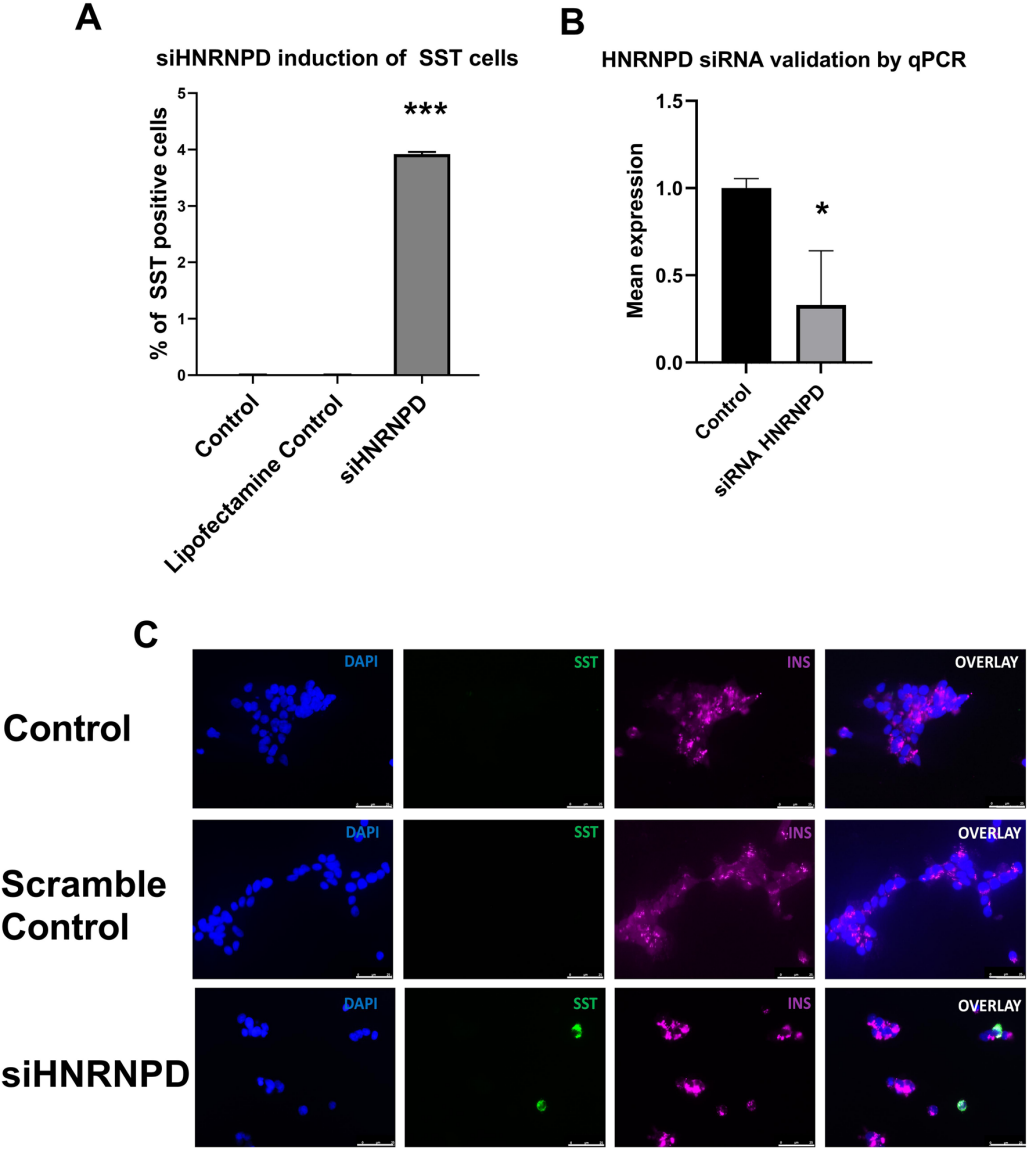
An siRNA against the *HNRNPD* gene induces the emergence of a somatostatin-positive cell population in un-stressed EndoC-βH1 clonal beta cells. **A** The percentage of somatostatin-positive cells that emerge in a clonal population of EndoC-βH1 cells treated with an siRNA against *HNRNPD* versus scrambled lipofectamine control is given on the Y axis, and the nature of treatment is given on the X axis. ***p =  < 0.0001. Error bars represent SD. **B** Validation of siRNA mediated *HNRNPD* gene knockdown by qRTPCR. The identity of the treatment (siRNA or scramble control) is given on the X axis, and the mean expression of *HNRNPD* is given on the Y axis. *p =  < 0.05. Error bars represent SD. **C** Immunofluorescence demonstrating hormone expression in EndoC-βH1 cells treated with a siRNA against *HNRNPD* or with scrambled transfection controls. EndoC-βH1. The top images left to right are controls without any treatment. The middle images left to right are of scramble lipofectamine controls. The bottom images left to right are of the siRNA against HNRNPD. Cell nuclei are labelled with DAPI (blue). Somatostatin (SST) expression is given in green. Insulin (INS) expression is given in magenta. Scale bars represent 0–25 μM. All statistical tests were performed using ANOVA

**Fig. 4:**
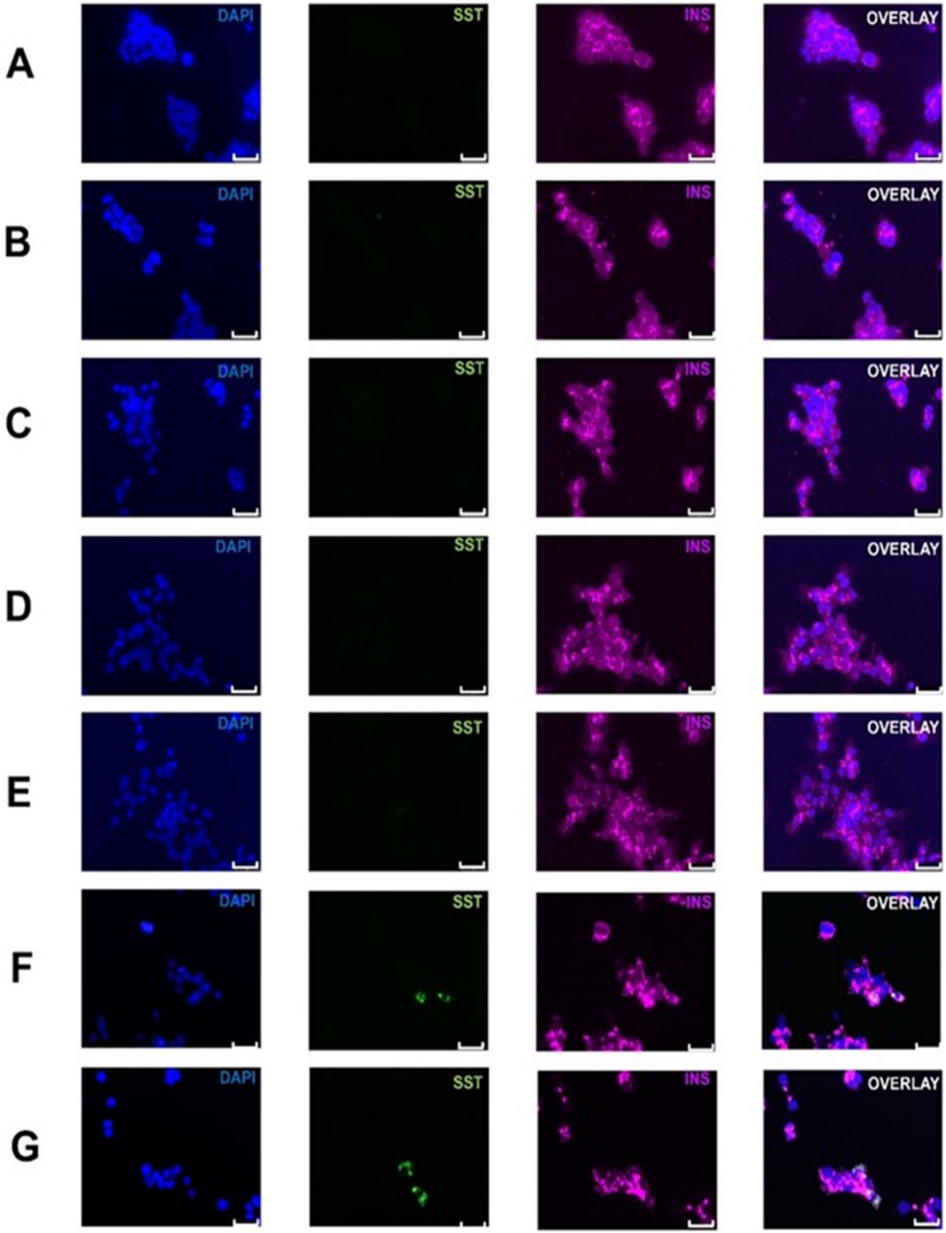
siRNA knockdown of additional coding genes from transcriptome data. The endocrine hormone profiles in EndoCβH1 cells with attenuated expression of the most dysregulated coding genes seen in transdifferentiated cells are presented here. Where genes demonstrated reduced expression in somatostatin-positive cells, we have ablated their expression with siRNA technology and assayed for the appearance of somatostatin positivity in non-stressed cells. Where target genes were upregulated in transdifferentiated cells, we have ablated their expression in stressed cells, and assayed for rescue. **A** *PHF12,* (down-regulated in transdifferentiated cells), **B** *C1ORF123,* (down-regulated in transdifferentiated cells), **C** *SENP7,* (down-regulated in transdifferentiated cells), **D** *DNAJC11*, (down-regulated in transdifferentiated cells), **E** *ZNF248*, (down-regulated in transdifferentiated cells), **F** *MALT1*, (up-regulated in transdifferentiated cells), **G** *GPN1,* (up-regulated in transdifferentiated cells). Nuclei are marked with DAPI in blue, insulin (INS) is given in pink, somatostatin (SST) is given in green. Scale bars represent 0–25 μM

To gain insight into the role of *HNRNPD* in altered beta cell fate, EndoC-βH1 cells were treated with a specific siRNA targeted to exon 3 of the *HNRNPD* gene, which targets all its known isoforms. We were able to reduce *HNRNPD* expression by 67% expression (*p* = 0.002). We found cells in which *HNRNPD* expression had been reduced but which had not been exposed to any cellular stress showed a gain of somatostatin expression in ~ 4% of the culture, comparable to our previous findings in stressed cells (n = 4 *p* = 1.66 × 10^–12^; Fig. 3). There was no evidence of a gain in somatostatin expression in either the control or lipofectamine control cultures. Although validation of the siRNA knockdowns for *PHF12, C1orf123* (*CZIB*), *DNAJC11, GPN1, MALT1, SENP7* and *ZNF248* and the expressed miRNAs also showed reduced expression of their target genes (Additional file 1: Table S3), there was no evidence for changes in differentiation status for any other target (Fig. 4 and Additional file 1: Figure S2).

## Discussion

In our previous work, we have described the emergence of a cell population exhibiting a gain in somatostatin expression, in otherwise clonal beta cells treated with multiple cellular stressors, a finding consistent with the elevated numbers of delta cells we have observed in the islets of patients with either T1D or T2D [14]. Changes in beta-cell differentiation states have also been reported by others, with dedifferentiation, re-differentiation and transdifferentiation to other pancreatic endocrine cell types observed [12–14]. Here, we present a comprehensive characterisation of the transcriptome of the somatostatin-positive cell population emergent in stressed clonal EndoC-βH1 beta cells, and demonstrate that the major changes present in these cells are apparent dysregulation of the non-coding genome, although for the technical reasons stated it was not possible to experimentally validate these changes. Finally, we implicate the RNA binding protein heterogeneous nuclear ribonucleoprotein particle D (HNRNPD) in the emergence of somatostatin positivity.

HNRNPD (also known as AUF1) is a multifunctional RNA binding protein (RBP) with roles in regulation of alternative splicing, mRNA transcription, RNA stability, RNA localisation and regulation of target transcript translation [16]. It, like other RNA binding proteins, has documented involvement in cellular stress response and is regulated by many cellular stresses, such as inflammation [22, 23]. Cross-regulatory relationships with inflammation also exist; many cytokines are destabilised by binding of HNRNPD to A-rich elements (AREs) in their 3ʹ untranslated regions [24]. Cytokine-induced *HNRNPD* expression has been linked with increased rates of beta cell apoptosis in patients with diabetes [19], while reduced *HNRNPD* expression is linked to survival of pancreatic beta cells [25]. Reduced expression of *HNRNPD* has been reported in the blood of children with T1D and is thought to be associated with disease progression [26]. Finally, HNRNPD has also linked with senescence [27], which is an emerging new driver of both T1D and T2D [28, 29]. This is probably mediated by the role of HNRNPD in telomere maintenance [30] and its contribution to splicing regulation in the context of cellular stress response [20, 22]. HNRNPD also has documented roles in differentiation decisions in other tissues; it plays a pivotal role in myogenesis by regulating fate-determining checkpoint mRNAs [31]. It plays a similar role in epithelial-mesenchymal transition through stabilisation of EMT transcription factors such as *TWIST1*, *SNAIL1 and ZEB1* [32, 33]. Interestingly, *HNRNPD* is a known downstream target of FOXO1 [15], which has previously implicated in beta cell identity decisions [8].

The changes to the non-coding transcriptome we observe in somatostatin-positive cells are entirely consistent with the consequences of dysregulation of *HNRNPD*. The majority of dysregulated transcripts we observe derive from the non-coding genome including precursor miRNAs and other small RNAs. HNRNPD is known to have roles in many features of miRNA biology, including miRNA biogenesis, as well as RISC loading and targeting to substrate [16], and long non-coding RNA transcripts (lncRNAs) are also targets of HNRNPD [34]. The GO pathways that demonstrate evidence of enrichment in WGCNA modules associated with somatostatin positivity are reflective of this. Several of the enriched pathways involve ubiquitination, which is tightly linked to ARE-mediated mRNA decay [35]. Pathways involved with with miRNA biogenesis and processing are also evident (see Table 3b). In accordance with the role of HNRNPD as a regulator of splicing, we also observe major dysregulation of mRNA processing. 26% of splicing events display differences in somatostatin-positive cells. The changes we have noted are mainly alternative 5ʹ or 3ʹ splice site usage or intron inclusion, rather than alteration in cassette exon usage. This is probably reflective of the role of HNRNPD in splice site silencing; the changes involve predominantly consensus splice sites, rather than the generation of truly aberrant transcripts from mid-exon splicing events. The GO pathways in which the genes displaying disrupted splicing are enriched are diverse, rather than reflecting specific changes in clustered groups of pathways as we see for the total gene expression changes. Many of the transcriptomic changes we have identified may therefore represent downstream effects of HNRNPD dysregulation, but at the present time, it is not possible to determine which of these changes are on the causal pathway to gain of somatostatin positivity, which are effects of this and which are effects of dysregulated *HNRNPD* expression, but unrelated to cell identity. These data are in agreement with our previous findings, as we have previously reported a downregulation of *HNRNPD* expression in bulk cultures of EndoC βH1 in response to cellular stressors [14]. We did not carry out a full assessment of effects on the whole transcriptome of stressed beta cells in our previous work however, so we would not have been able to identify changes to the non coding genome in our earlier studies. A limitation of this study is the technical difficulty of reduced RNA integrity from FACS sorted cells, which precluded subsequent qPCR validation of gene expression and may have influenced miRNA detection. However, since both somatostatin-positive and somatostatin negative cell populations had been subjected to the same treatment and sorted from the same sample it is unlikely that this significantly skewed these data. We also note that knock down of *HNRNPD* using an siRNA providing best coverage for the gene rather than using multiple different siRNAs may increase the likelihood for off target effects, although the siRNA in question was supplied as a validated siRNA by the supplier.

This work provides evidence for a role for HNRNPD in the stress-related determination of beta cell identity. Our work is consistent with a model whereby the cellular stresses induced by disrupted metabolic homoeostasis may lead to the dysregulation of *HNRNPD* expression and consequent changes to the non-coding genome and patterns of alternative splicing and mRNA turnover. Changes in cell identity may therefore represent a stress evasion mechanism adopted by beta cells, or it may reflect a chance event. Several questions remain. Firstly, the changes in hormone expression may not indicate full transdifferentiation. It is more likely that the cell identity changes are partial, since the cells do not express of markers of delta cell fate such as *HHEX* or *GHSR*. Secondly, it is not yet clear which of the downstream changes in *HNRNPD* target genes drive the changes in cell identity we have observed, and also why only a small proportion of clonal beta cells gain somatostatin-positivity. This may indicate that some cells in an otherwise clonal population have some characteristic that renders them more prone to changes in hormone expression; beta cells are known to differ in their characteristics; some beta cells in pancreatic islets are ‘hub’ cells which are more sensitive to insult [36], and even a clonal cell line is rarely completely clonal. Small numbers of somatostatin-positive cells have previously been reported by others in untreated EndoC-βH1 [37], but this may reflect the general sensitivity of this cell line to cellular stress if not carefully controlled. We have not observed this phenomenon in any of our untreated cell populations. Another possibility is that the heterogeneity in cell identity response may reflect a stochastic aspect of non-coding dysregulation; some combinations of non-coding dysregulation may result in changes in hormone profile, whilst others do not. Finally, EndoC βH1 cells are derived from foetal, rather than adult, pancreatic tissue and so may be more prone to changes in their differentiation state, since they have not undergone the final stages in their development.

## Conclusion

The molecular mechanisms by which RNA binding proteins contribute to determination of cell identity is an important area of study. The data presented here are consistent with a model whereby stress-related changes in *HNRNPD* gene expression lead to transcriptome-wide changes to the dynamics of RNA regulation, dysregulation of the non-coding genome and the subsequent emergence of a somatostatin-positive cell population in the human pancreatic beta cell line EndoC βH1. Studies have shown that even in long duration diabetes, a proportion of glucose responsive beta cells persist [4]. Protection of this remaining beta cell mass is an important goal, even in long duration diabetes. Understanding the processes by which beta cell mass is lost may provide a key to protection of this remaining beta cell reservoir. This role for RNA binding proteins is an emerging area of research and further studies will be needed to fully elucidate the interactions between *HNRNPD* and the non-coding genome in cell fate decisions.

## Materials and methods

### Culture and treatment of EndoC βH1 cells

The human clonal beta cell line EndoC-βH1 (passage < 25) was seeded in T25 flasks at a density of 1.75 × 10^6^ cells/mL and maintained according to a modified humanised culture protocol as previously described for 72 h prior to treatment with diabetes-related cellular stressors [38]. For assessment of the effects of elevated glucose and altered lipids (glucolipotoxicity), cells were treated with a cocktail of 25 mM d-glucose and 50 μM palmitic acid for 48 h. Controls were maintained in 5 mM glucose media. Each treatment was carried out in six biological replicates, along with ethanol vehicle-only controls.

### Enrichment for somatostatin-positive populations of human EndoC-βH1 cells

The small (~ 5%) sub-population of somatostatin-positive EndoC-βH1 cells was isolated by FACS with the BD Bioscience FACS Aria III (BD Biosciences, San Jose, USA), using an intracellular antibody against somatostatin (Alexa Fluor 488 mouse anti-human somatostatin, 1:100 dilution, clone U24-354, 566032, BD Biosciences, San Jose, USA). Cells were fixed using ice-cold MeOH added dropwise and the cell membrane was permeabilised using 0.02% triton. FACS DIVA software 4-32-16 purity filter was used to ensure sample purity and gated as FCS/SSC/FITC. Doublet cells were removed to ensure single cell populations and sorted cells were run back through to check for contamination. Approximately 20,000 cells were collected from six separate cultures into 750 μL Tri reagent LS (T3934, Sigma Aldritch, Steinheim, Germany). The total volume was adjusted to 1 mL using DPBS (Thermo Fisher, Foster City, USA) and RNA was purified using a column based Zymo Direct-Zol RNA Miniprep kit (Cambridge Bioscience, Cambridge, UK) according to the manufacturer’s instructions to maximise yield.

### Differential gene expression analysis of somatostatin-positive cells relative to somatostatin-negative cells

We measured patterns of total gene expression and transcriptome wide patterns of alternative splicing in six separate biological replicates of somatostatin-positive and somatostatin-negative cell populations using Clariom D pico GeneChip Whole Transcriptome (WT) expression arrays (Thermo Fisher, Waltham, MA, USA). This technology was employed because the cell sort methodology with an internal cell marker precludes the extraction of full length, intact RNA for analysis with RNAseq or similar approaches, and necessitates alternative technologies for the assessment of the transcriptome. The Clariom D Pico approach allows the assessment of transcriptomic changes in RNA that has undergone considerable degradation [39, 40], RNA integrity was assessed as part of the Clariom D pico library preparation and passed quality and integrity checks (UK Bioinformatics, King’s College, London, UK). Data underwent quality control for probeset mean for hybridisation intensity, probeset residual mean which compares probeset signal to residual signal, poly-A positive spike in controls as control genes and positive versus negative area under the curve. SST-RMA was selected to reduce background and normalize intensity following which a differential expression analysis was undertaken using the Transcriptomic Analysis Console (TAC) (Applied Biosystems) software (TAC 4.0.2.10) designed for the analysis of Clariom D Pico data, using the default settings. Differential gene expression in TAC uses the Limma algorithm followed by EventPointer for exon level analysis [21, 41].

### Identification of differentially-expressed modules of genes in somatostatin-positive cells

Although we detected some genes with altered expression in somatostatin-positive cells which were nominally significant, but did not meet the Benjamini Hochberg threshold for multiple testing. We therefore employed Weighted Gene Co-expression Network Analysis (WGCNA) [42] to identify potentially co-regulated modules of total gene expression in our dataset. Data were initially filtered to remove ‘AceView’ predicted transcripts and unlabelled genes. Genes with the same gene symbol were aggregated into a single entry with the average expression of each individual probe. We next inferred and clustered the co-expression networks using a soft threshold power of 12, and a minimum module size of 50. Module eigengenes were calculated using a module eigengene dissimilarity threshold of 0.25. Finally, module eigengenes were used to infer module differential co-expression between the two conditions and taking into account the paired design of the study. Genes from within the top three clusters were taken forward to Panther gene ontology (GO) biological processes analysis [43, 44] to identify the biological or functional pathways enriched in differentially-regulated genes.

### Assessment of genes demonstrating differential expression in somatostatin-positive cells by gene class

We carried out an enrichment analysis of the distribution of coding gene, multiple complex loci (locus contains more than one gene type e.g. ribosomal and noncoding), non-coding gene, small RNA, precursor microRNA and ribosomal RNA gene distribution in somatostatin-positive and somatostatin-negative cells using the χ^2^ statistic. The expected number of genes within a class was determined by taking the total number of genes in each class for the entire gene chip array. The observed number of genes in each class with a p value of < 0.05 was then determined and compared to the expected value by χ^2^ analysis for each gene class.

### Validation of differentially expressed miRNAs

We selected a number of non-coding genes with expression patterns suggestive of differential expression for further validation. The need to permeabilise the cell membrane for antibody entry for the FACS however rendered the RNA from the sorted cells unsuitable for qRTPCR analysis of coding genes and other long transcripts. However, the RNA was of good enough quality to allow validation of some small RNAs. The top 100 most dysregulated transcripts identified nine differentially-expressed precursor microRNAs (pre-miRNAs) in somatostatin-positive cells which we were able to validate differential effects on their mature miRNA products by qRTPCR which were analysed using ANOVA. These were the precursors to miR-373, miR-543, miR-548t, miR-181a-3p, miR-181a-5p, miR450a-1-3p, miR-450a-5p, miR-6874-3p and miR-6874-5p. We synthesised cDNA from RNA extracted from six separate biological replicates for the somatostatin-positive and somatostatin-negative populations of cells using the TaqMan advanced microRNA cDNA synthesis kit (Thermo Fisher, Waltham, MA, USA). We then performed qPCR validation of the mature microRNAs, using TaqMan advanced microRNA assays (Thermo Fisher, Waltham, MA, USA). Reactions were carried out in three biological replicates and three technical replicates with endogenous controls (miR-106b-3p, miR-93-5p and miR-27b-3p) selected for stability. qRTPCR reaction mixes comprised 2.5 μL Taqman^®^ Universal PCR Mastermix II (no AmpErase^®^ UNG) (Thermo Fisher, Waltham, MA, USA), 1.75 μL dH_2_O, 0.5 μL cDNA and 0.25 μL Taqman^®^ gene assay (Thermo Fisher, Foster City USA) in a 5 μL reaction volume. Cycling conditions were: 50 °C for 2 min, 95 °C for 10 min and 50 cycles of 15 s at 95 °C for 30 s and 1 min at 60 °C. Assay identifiers are given in Additional file 1: Table S1.

### In vitro manipulation of differentially-expressed non-coding transcripts

For pre-miRNAs where we had found evidence that their corresponding mature transcripts demonstrated dysregulated expression in somatostatin-positive cells (miR181a-5p and miR-543), we carried out an in vitro evaluation of their ability to abrogate (in stressed cells) or induce (in non-stressed cells) the emergence of a somatostatin-positive cell population. EndoC-βH1 cells at passage < 25 were seeded into six well plates at a density of 1 × 10^6^ cells/mL and maintained until 70% confluence. MirVana mimics and antagomiRs to miR-543 and miR-181a-5p were purchased from Thermo Fisher (Waltham, MA, USA). To assess whether recapitulating the miR levels seen in stressed EndoC-βH1 cells caused the emergence of a somatostatin-positive cell population, we introduced a mimic to miR-543 or an antagomiR to miR-181a-5p into EndoC-βH1 cells at a concentration of 75 pmol using Lipofectamine 3000 (Thermo Fisher, Waltham, MA, USA) for 48 h. To determine whether recapitulating the miR levels in non-stressed EndoC-βH1 cells treated with 25 mM glucose and 50 μM palmitic acid, we introduced a mimic to miR-181a-5p, or an antagomiR to miR-543. Untreated and scrambled controls were also included in the experiment to control against non-specific effects of transfection. After 48 h, RNA was extracted using Tri-Reagent (Thermo Fisher, Waltham, USA), and qRTPCR was carried out for assessment of target knockdown or mimic efficiency. As previously described, cDNA was synthesized using the TaqMan advanced microRNA cDNA synthesis kit (Thermo Fisher, Waltham, MA, USA). For qPCR validation of microRNA manipulations we used TaqMan advanced microRNA assays (Thermo Fisher, Waltham, MA, USA) for three biological replicates and three technical replicates with the same endogenous controls previously described. qRTPCR reaction mixes comprised 2.5 μL Taqman^®^ Universal PCR mastermix II (no AmpErase^®^ UNG) (Thermo Fisher, Waltham, MA, USA), 1.75 μL dH_2_O, 0.5 μL cDNA and 0.25 μL Taqman^®^ gene assay (Thermo Fisher, Foster City USA) in a 5 μL reaction volume. Cycling conditions were: 50 °C for 2 min, 95 °C for 10 min and 50 cycles of 15 s at 95 °C for 30 s and 1 min at 60 °C. As previously, expression differences and cell count were analysed using ANOVA. Identifiers are given in Additional file 1: Table S2.

### In vitro manipulation of differentially-expressed coding transcripts

We carried out an in vitro manipulation of levels of the eight coding genes represented in the top 100 dysregulated genes and demonstrating > twofold increase or decrease in expression in the somatostatin-positive enriched cell population (*HNRNPD*, *PHF12*, *C1orf123*, *ZNF248*, *SENP7*, *DNAJC11, MALT1* and *GPN1*) using targeted gene knockdown. Experiments were carried out in EndoC-βH1 cells in three biological replicates using validated Silencer siRNAs selected for providing best coverage for the target gene. (Thermo Fisher, Waltham, USA). Assay Identifiers are given in Additional file 1: Table S3. Where we had observed genes to be down-regulated in somatostatin-positive cells, we knocked down gene expression in non-stressed cells and assayed for the emergence of a somatostatin-positive cell population. Where we had found genes to be up-regulated in the somatostatin-positive cells, we knocked down target expression in stressed cells (25 mM glucose, 50 μM palmitic acid), and assayed for ablation of the somatostatin-positive population. As before, EndoC-βH1 cells at passage < 25 were seeded into 6 well plates at a density of 1 × 10^6^ cells/mL and maintained until 70% confluence. siRNAs were introduced at a concentration of 75 pmol per transfection using Lipofectamine 3000. Untreated and scramble-treated cells were also included to control for effects induced by the transfection process itself. After 48 h, RNA was extracted using Tri-Reagent (Thermo Fisher, Waltham, USA) for assessment of target knockdown efficiency. For validation of the knockdowns, all reactions were carried out in three biological replicates and three technical replicates. cDNA synthesis was carried out using EvoScript universal cDNA master (Roche Life Science, Burgess Hill, UK) according to manufacturer’s instructions. The endogenous control genes *PPIA, IDH3B, HPRT1 and GUSB* were empirically selected for stability and, as previously, qRTPCR reaction mixes were 2.5 μL Taqman^®^ Universal PCR mastermix II (no AmpErase^®^ UNG) (Thermo Fisher, Waltham, MA, USA), 1.75 μL dH_2_O, 0.5 μL cDNA and 0.25 μL Taqman^®^ gene assay (Thermo Fisher, Foster City USA) in a 5 μL reaction volume. The cycling conditions were: 50 °C for 2 min, 95 °C for 10 min and 50 cycles of 15 s at 95 °C for 30 s and 1 min at 60 °C. As before, statistical analysis was carried out by ANOVA.

### Assessment of effects of in vitro gene manipulation on beta cell identity

To assess the effect of targeted manipulation of miRNA or coding gene targets, we assessed the hormone profile of treated EndoC-βH1 cells by immunofluorescence. Cells were fixed using 4% paraformaldehyde for 15 min at 4 °C. Primary antibodies to SST were diluted in phosphate buffered saline (PBS) with 0.1 M Lysine, 10% donor calf serum, 0.02% sodium azide and 0.02% Triton X100 (ADST), to permeabilise the cell membranes and incubated overnight at 4 °C. Primary antibodies were visualised using species specific highly cross-absorbed secondary antibodies (Abcam, Cambridge, UK) diluted in ADST at 1/400 and incubated for 1 h at room temperature. Sequential staining was then performed with an antisera raised against insulin (Additional file 1: Table S4) diluted in ADST for 1 h, followed by a goat anti guinea-pig Alexa Fluor 555 along with DAPI (Sigma-Aldrich; 1 μg/mL) for 1 h. Slides were visualised using a Leica AF6000 microscope (Leica, Milton Keynes, UK) and processed using the standard LASX Leica software platform. Ten randomly selected images were taken for each of the three biological replicates. Details of all antibodies used are provided in Additional file 1: Table S4.

## Supplementary Information

**Additional file 1: Table S1.** Quantification of mature miRNAs in stressed EndoC βH1 beta cells. **Table S2**. Mirna Mimic/Antagomir Treatments In Endoc Βh1 Cells. **Table S3.** quantitative pcr validation of sirna-silenced coding genes demonstrating dysregulation in ‘transdifferentated’ cells. **Table S4. **Details of antibodies and experimental conditions. **Figure S1.** Violin plot of the proportion of somatostatin (SST) positive cells following treatment with miRNA mimics/inhibitors or siRNAs to coding genes. **Figure S2.** Violin plot of the proportion of somatostatin (SST) positive cells following treatment with siRNAs to coding genes dysregulated in somatostatin positive cells.

## Data Availability

The data pertaining to this work is available from GEO under the Accession GSE173423.
