# Supplementary material for: Changes to the identity of EndoC-βH1 beta cells may be mediated by stress-induced depletion of HNRNPD
Source: Cell Biosci. 2021 Jul 23;11:144. doi: 10.1186/s13578-021-00658-6 (PMC8305497; doi:10.1186/s13578-021-00658-6)
Supplement: Supplementary file 1 — Additional file 1: Table S1. Quantification of mature miRNAs in stressed EndoC βH1 beta cells. Table S2. Mirna Mimic/Antagomir Treatments In Endoc Βh1 Cells. Table S3. quantitative pcr validation of sirna-silenced coding genes demonstrating dysregulation in ‘transdifferentated’ cells. Table S4. Details of antibodies and experimental conditions. Figure S1. Violin plot of the proportion of somatostatin (SST) positive cells following treatment with miRNA mimics/inhibitors or siRNAs to coding genes. Figure S2. Violin plot of the proportion of somatostatin (SST) positive cells following treatment with siRNAs to coding genes dysregulated in somatostatin positive cells. [file 13578_2021_658_MOESM1_ESM.docx]

***Changes to the identity of EndoC -βH1 beta-cells may be mediated by stress-induced depletion of HNRNPD.***

*Nicola Jeffery^1^, David Chambers^2^, Brandon M. Invergo^3^, Ryan M. Ames^4^, Lorna W. Harries^1^.*

*^1^Institute of Biomedical and Clinical Sciences, University of Exeter Medical School, Barrack Road, Exeter, UK, EX2 5DW*

*^2^Wolfson Centre for Age Related Diseases, Kings College London, London, UK, WC2R 2LS*

*^3^ Translational Research Exchange @ Exeter, University of Exeter, Stocker Road, Exeter EX4 4QJ*

*^4^ Biosciences, University of Exeter, Stocker Road, Exeter, EX4 4QD*

**Corresponding author**

Prof L.W. Harries

Institute of Biomedical and Clinical Studies

University of Exeter Medical School

Barrack Road

Exeter

EX2 5DW

Tel: 01392 406779

Email: [L.W.Harries@exeter.ac.uk](mailto:L.W.Harries@exeter.ac.uk)

ORCID : 0000-0001-7791-8061

# Contents

[Contents 2](#_Toc70326148)

[Table S1. Quantification of mature miRNAs in stressed EndoC βH1 beta cells. 3](#_Toc70326149)

[Table S2. Mirna Mimic/Antagomir Treatments In Endoc Βh1 Cells. 3](#_Toc70326150)

[Table S3. quantitative pcr validation of sirna-silenced coding genes demonstrating dysregulation in ‘transdifferentated’ cells. 4](#_Toc70326151)

[Table S4. Details of antibodies and experimental conditions. 4](#_Toc70326152)

[Figure S1. Violin plot of the proportion of somatostatin (SST) positive cells following treatment with miRNA mimics/inhibitors or siRNAs to coding genes. 5](#_Toc70326153)

[Figure S2. Violin plot of the proportion of somatostatin (SST) positive cells following treatment with siRNAs to coding genes dysregulated in somatostatin positive cells.. 6](#_Toc70326154)

# Table S1. Quantification of mature miRNAs in stressed EndoC βH1 beta cells.

The assay identifiers for the mature miRNAs measured in EndoCβH1 cells are given here, along with information on whether the mature forms were expressed in somatostatin-enriched (n = 3) or insulin-enriched (n = 3) EndoCβH1 cells, and if so, whether there were differences in their expression between these two cell types. P values as determined by Students t test are presented, below along with mean difference in expression for differentially regulated miRNAs.

| **microRNA** | **Assay ID** | **Expressed INS** | **Expressed SST** | **p value** | **Mean diff** |
| --- | --- | --- | --- | --- | --- |
| **miR-373** | 478073_miR | No | No | *n/ a* |  |
| **miR-548ac** | 479005_miR | No | No | *n/ a* |  |
| **miR-548t-3p** | 479004_miR | No | No | *n/ a* |  |
| **miR-450a-1-3p** | 478911_miR | No | No | *n/ a* |  |
| **miR-450a-5p** | 478106_miR | No | No | *n/ a* |  |
| **miR-181a-3p** | 479405_miR | No | No | *n/ a* |  |
| **miR-6874-3p** | 480498_miR | No | No | *n/ a* |  |
| **miR-6874_5p** | 480499_miR | No | No | *n/ a* |  |
| **miR-181a-5p** | 477857_miR | Yes | Yes | *0.001* | *0.468* |
| **miR-543** | 478155_miR | Yes | Yes | *0.001* | *-0.934* |

Table S2. miRNA mimic/antagomiR treatments in EndoC βH1 cells. The assay identifiers for the miRNA mimics and miRNA antagomiRs used in this work are presented below. The percentage change in miR-543 and miR-181a expression between miRNA mimic or AntagomiR-treated EndoCβH1 beta cells and vehicle-only controls (n= 3 each) is given, as well as the 95% confidence intervals (95% CI), the standard error of the difference (SED) and the *p* values of effect, as determined by Student’s t test.

| **microRNA names** | **Assay IDs** | ***p* value** | **% diff.** | **SED** | **95% CI** | |
| --- | --- | --- | --- | --- | --- | --- |
|  |  |  |  |  | **Lower** | **Upper** |
| **miR-543 mimic** | MC13037 | 0.017 | + 33.63% | 1.16 | 0.865 | 6.364 |
| **miR-543 inhibitor** | MC13037 | 0.001 | - 42.12% | 1.302 | -10.252 | -4.092 |
| **miR-181a-5p mimic** | MH10421 | 0.004 | + 32.02% | 0.599 | 1.061 | 3.897 |
| **miR-181a-5p inhibitor** | MH10421 | 0.018 | - 34.17% | 1.063 | -5.596 | -0.768 |

Table S3. Quantitative PCR validation of siRNA-silenced coding genes demonstrating dysregulation in ‘transdifferentated’ cells.

The assay identifiers for the genes targeted by the siRNAs used in this work are given below. The percentage change in expression, the 95% confidence intervals of effect, the standard error of the difference (SED) and the p value as determined by students t test are presented.

|  | **siRNA IDs** | **p value** | **% Diff.** | **Std. Error Difference** | **95% Confidence Interval of the Difference** | |
| --- | --- | --- | --- | --- | --- | --- |
|  |  |  |  |  | Lower | Upper |
| ***HNRNPD*** | s6723 | 0.002 | - 66.25% | 0.074 | -1.037 | -0.549 |
| ***PHF12*** | s33518 | 0.03 | - 80.37% | 0.488 | -2.967 | -0.26 |
| ***C1ORF123*** | s29927 | 0.014 | - 74.36% | 0.179 | -1.243 | -0.249 |
| ***DNAJC11*** | s31371 | 5.77 x 10^-8^ | - 88.06% | 0.0169 | -1.759 | -1.665 |
| ***GPN1*** | s22323 | 1.11 x 10^-4^ | - 86.61% | 0.098 | -1.755 | -1.211 |
| ***MALT1*** | s21398 | 0.005 | - 78.41% | 0.072 | -1.247 | -0.642 |
| ***SENP7*** | s32960 | 0.001 | - 89.38% | 0.194 | -2.707 | -1.63 |
| ***ZNF248*** | s32889 | 0.001 | - 85.75% | 0.119 | -1.516 | -0.855 |

Table S4. Details of antibodies and experimental conditions.

Details of the antibodies used in this study, the lot numbers, the species origin and the dilution factors used are given here.

| **Antibody** | **Supplier** | **Cat #** | **Lot number/ Clone** | **Species** | **Concentration used:** |
| --- | --- | --- | --- | --- | --- |
| INS | DAKO | 80564 | 10088287 | Guinea-pig polyclonal | 1/363 |
| SST | Abcam | Ab30788 | GR213035-1 | Rat monoclonal IgG2b | 1/200 |
| SST | BD Biosciences | 566032 | U24-354 | Mouse IgG_2b_ | 1/100 |

**Figure S1. Violin plot of the proportion of somatostatin (SST) positive cells following treatment with miRNA mimics/inhibitors or siRNAs to coding genes.** Response of 25mM glucose and 50μM palmitic acid (GP) treated and untreated EndoC-βH1 cells to miRNA mimics or inhibitors to miR-543 or miR-181a-5p. Where the miRNA in question was upregulated in sorted cells, we assayed for ablation of somatostatin (SST) expression in GP stressed cells in response to treatment with a mimic. Where the miR was down regulated in sorted cells, we assayed for appearance of a SST +ve population in response to an inhibitor of the miRNA in question. The % SST positive cells in the culture is given on the Y axis and the treatment conditions on the x axis. **1** = GP control, **2** = GP + miR-543 mimic, **3** = untreated control, **4** = miR-543 inhibitor, **5** = GP control, **6** = GP + miR-181a-5p inhibitor, **7** = untreated control, **8** = miR-181a-5p mimic. All data are derived from 3 biological replicates.


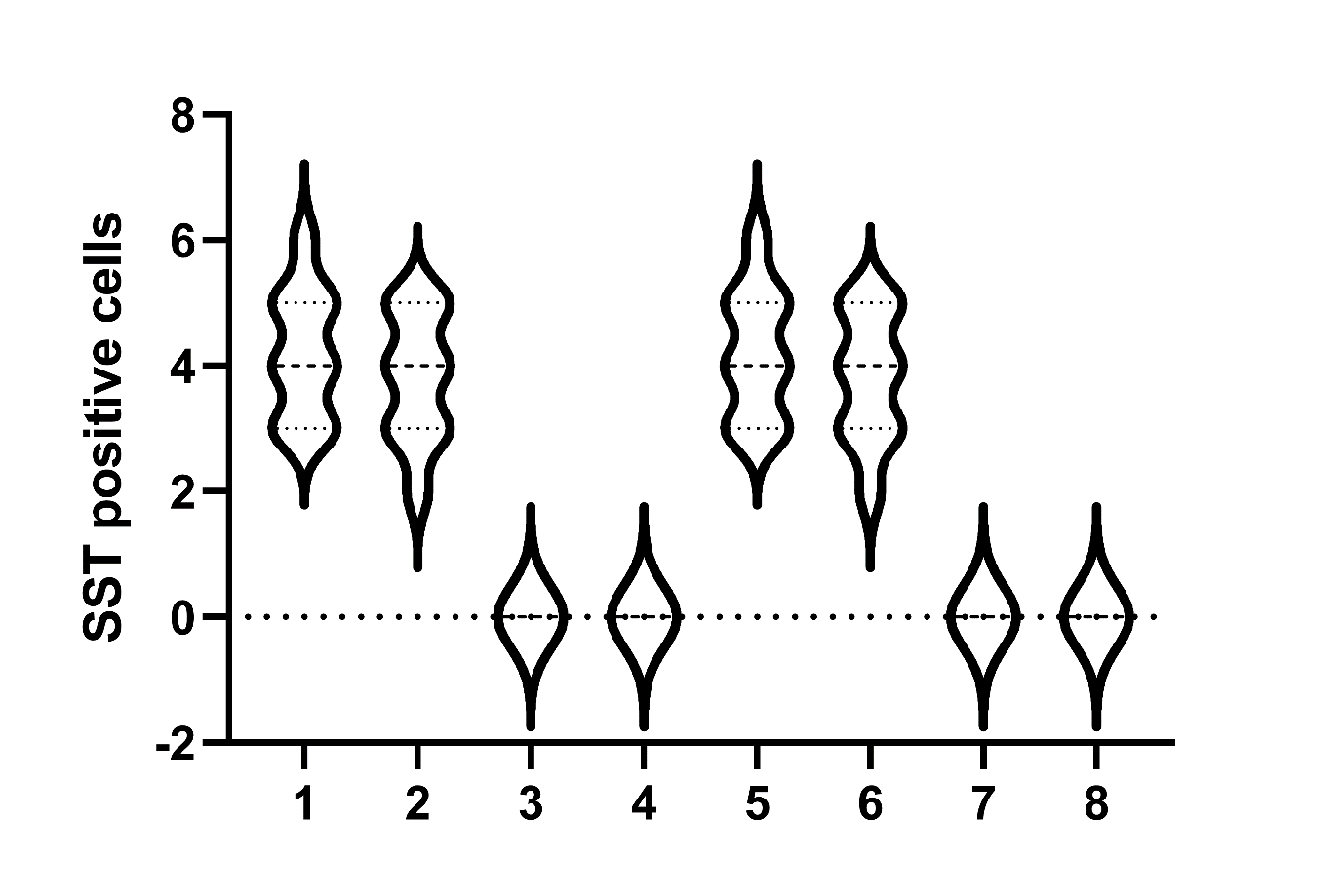


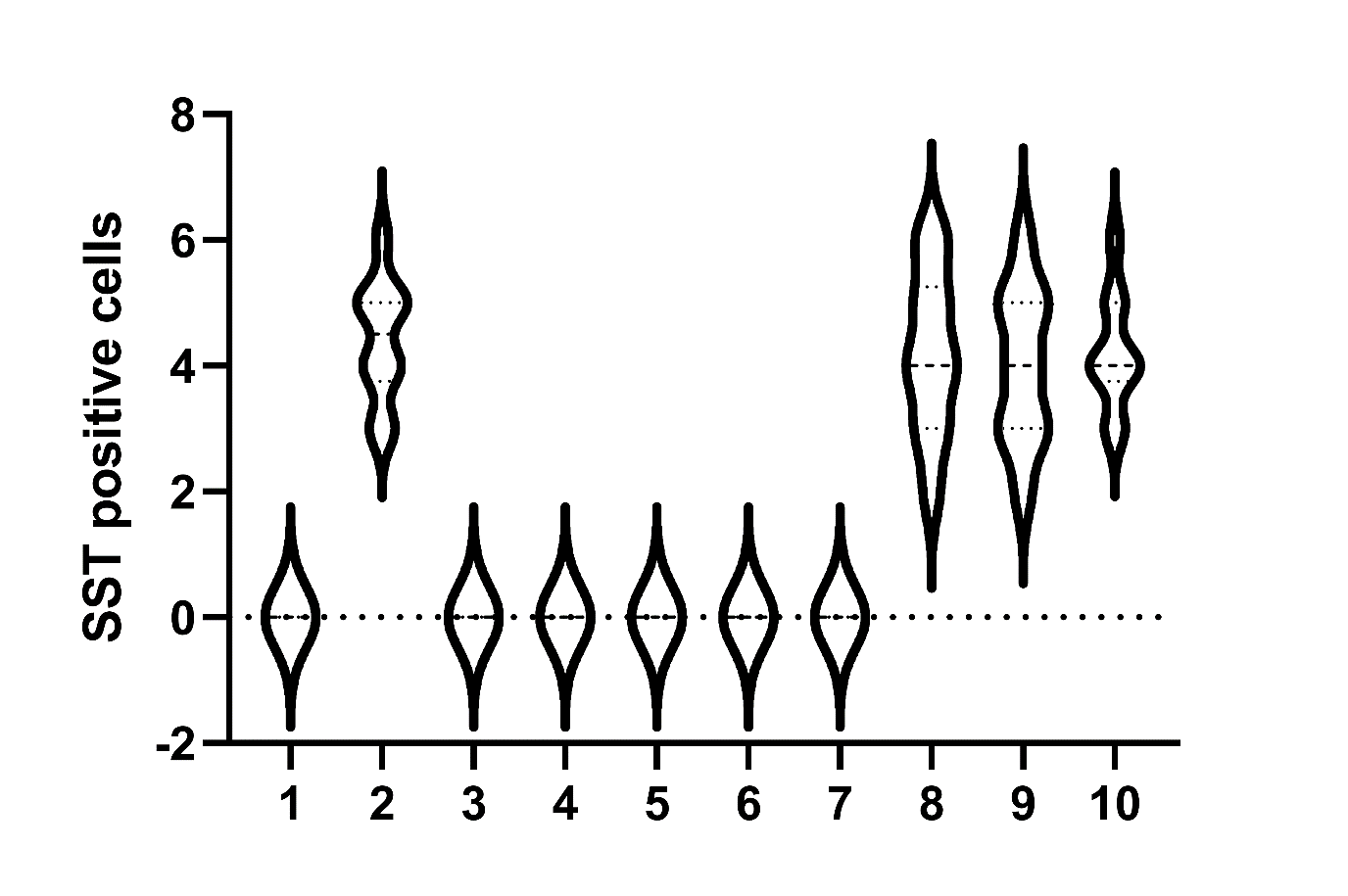
**Figure S2. Violin plot of the proportion of somatostatin (SST) positive cells following treatment with siRNAs to coding genes dysregulated in somatostatin positive cells.**  **B.** Response of 25mM glucose and 50μM palmitic acid (GP) treated and untreated EndoC-βH1 cells to siRNAs designed to genes dysregulated in sprted somatostatin (SST) positive cells. Where the gene in question was upregulated in sorted cells, we assayed for ablation of somatostatin (SST) expression in GP stressed cells in response to gene knockdown. Where the gene was down regulated in sorted cells, we assayed for appearance of a SST +ve population in response to siRNA inhibition of the gene in question. The % SST positive cells in the culture is given on the Y axis and the treatment conditions on the x axis. **1** = untreated control, **2** = siHNRNPD, **3** = siPHF12, **4** = siC1orf123, 5 = siSENP7, 6 = siDNAJC11, 7 = siZNF248, 8 = GP treated control, 9 = GP-treated siMALT1, 10 = GP treated siGPN1.
